# Mechanisms underlying the lung-protective effects of FLow- controlled EXpiration

**DOI:** 10.1186/cc13486

**Published:** 2014-03-17

**Authors:** S Schumann, U Goebel, J Haberstroh, J Guttmann

**Affiliations:** 1 University Medical Center Freiburg, Germany

## Introduction

During mechanical ventilation the expiration occurs passively and is determined by the recoil forces of the respiratory system. In an experimental study in pigs we could find that linearization of the expiratory flow via FLow-controlled EXpiration (FLEX) [1] is lung protective [2]. Utilizing electrical impedance tomography (EIT) we aimed at investigating the mechanisms underlying the lung-protective effects of FLEX.

## Methods

All experiments were approved by the local animal welfare committee. Twelve pigs with oleic acid-induced lung injury were ventilated in the volume-controlled mode (VCV). In six animals, expiratory flow was linearized via FLEX. PEEP was set to achieve similar mean airway pressure in the control group (*n *= 6) and in the FLEX group (*n *= 6). Using EIT, the local distribution of ventilation was measured and alveolar derecruitment during the no-flow phase in late expiration was quantified.

## Results

During ventilation with FLEX the no-flow phase in late expiration was reduced by 50% compared with passive expiration. Derecruitment during the no-flow phase was clearly reduced by FLEX compared with VCV. Furthermore, intratidal ventilation was more homogeneously distributed during ventilation with FLEX compared with conventional passive expiration.

## Conclusion

In comparison with conventional VCV with passive expiration, the no-flow phase in late expiration is reduced and so is the time the lung persists on the lowest pressure level (PEEP) during the breath. The reduced low-pressure time is associated with reduced end- tidal derecruitment. In a lung mechanically stabilized and recruited by sustained airway pressure throughout the expiration phase, the distribution of ventilation is more homogeneous. These mechanisms of alveolar recruitment maintenance can explain the lung-protective effects of FLEX.
